# Non-prescribed antimicrobial use and associated factors among customers in drug retail outlet in Central Zone of Tigray, northern Ethiopia: a cross-sectional study

**DOI:** 10.1186/s13756-017-0227-7

**Published:** 2017-06-26

**Authors:** Negash Hadera Gebrekirstos, Birhanu Demeke Workneh, Yosef Sibhatu Gebregiorgis, Kebede Haile Misgina, Negassie Berhe Weldehaweria, Meresa Gebremedhin Weldu, Hailay Siyum Belay

**Affiliations:** 1 Private pharmacy owner, Aksum, Ethiopia; 2 College of Health Sciences, Wollo University, Desie, Ethiopia; 3 Department of Public Health, College of Health Sciences, Aksum University, P.O.Box: 298, Aksum, Ethiopia

**Keywords:** Antimicrobial, Magnitude, Non-prescribed, Ethiopia, Drug retail outlets

## Abstract

**Background:**

Non-prescribed antimicrobial use and their resistance are among the main public health problems, worldwide. In Ethiopia, particularly in the northern part, the magnitude of non-prescribed antimicrobial use and its major determinants is not yet well known. Thus, this study was done to assess the magnitude of non-prescribed anti-microbial use and associated factors among customers in drug retail outlet in Central Zone, Tigray, Ethiopia.

**Methods:**

A drug retail outlet based cross-sectional study was conducted among adults aged 18 years and above. A multistage sampling procedure was used to select study participants. Data were collected using a structured questionnaire by druggists under the supervision of pharmacists. Data were entered into EpiInfo software version 3.5.4. Binary logistic regression was used to identify independently associated variables in bivariate and multivariable analyses using SPSS version 21. Odds Ratios with 95% confidence intervals were estimated.

**Results:**

From 829 study samples, a total of 780 respondents participated in this study with a response rate of 94.1%. Of 367 respondents who received non-prescribed antimicrobial, 249 (67.8%), 121 (33%), and 94 (25.6%) of them were males, secondary school and paid employed respectively. The magnitude of non-prescribed antimicrobial use was 47.1% (95% CI: 43.8, 50.5). The factors which were independently associated with non-prescribed antimicrobial use were male sex [AOR = 1.72, 95% CI = 1.21, 2.44], seeking modern health care in private/Non-Governmental Organization (NGO) [AOR =0.47, 95% CI; 0.23, 0.98], moderate waiting time in health care facilities [AOR = 1.92, 95% CI; 1.20, 3.09], delayed waiting time in health care facilities [AOR = 1.56, 95% CI; 1.03, 2.38], ever received antimicrobial [AOR = 3.51, 95% CI; 2.45, 5.02], and frequency of purchasing non-prescribed antimicrobial (1–3 times and 4 times, [AOR = 2.04, 95% CI; 1.36, 3.06] and [AOR = 2.66, 95% CI; 1.24, 5.68] respectively).

**Conclusion:**

The magnitude of non-prescribed antimicrobial use was high. Familiarizing with health care utilization and delayed waiting time in health care facilities were the very important factors independently associated with non-prescribed antimicrobial use. Emphasis should be given to community education through involvement of the private health sector and health care providers. Regulation and policy enforcement are also necessary to promote the rational use of antimicrobial.

## Background

Non-prescribed antimicrobial use refers to the use of medicines to treat self-diagnosed disorders without consulting a medical practitioner and any medical supervision [1]. These medicines are essential in medical health care system, especially in the resource scarcity settings where infectious diseases are common causes of deaths [2]. Low coverage in availability and accessibility of the healthcare delivery systems due to inequitable health care distribution, high costs, lack of health care professionals, unregulated and unmonitoring distribution of medicines, patient attitudes towards physicians are some of the key drivers of misuse of anti-microbials in the resource scarce settings [3, 4].

Globally, non-prescribed antimicrobial drugs use even for non-bacterial diseases are the most commonly practiced or oversight by health-care professionals [5, 6]. The immediate consequences of non-prescribed antimicrobial drugs use include; short duration of treatment, inadequate dose, sharing of medicines, stopping treatment upon the improvement of disease symptoms [7]; adverse drug reactions and masking of underlying infectious processes [3, 5, 8].

As a result, antimicrobial-resistance is a major emerging global public health problem in communities with frequent non-prescribed antimicrobial use [5, 9, 10]. World Health Organization (WHO) in 2014 examined that anti-microbial resistance, in particular, antibacterial resistance has health and economic burden [10]. Nowadays, non-prescribed antimicrobial use is widely affected by improvement in people’s education, general knowledge and socio-economic status [1]. In Low and Middle Income Countries (LMICs), educational interventions, improving access to quality of public healthcare, and enforcement of regulations on non-prescribed medicine use could help mitigate the challenge of antimicrobial use and resistance [5, 11].

A study in developing countries has evidenced that the magnitude of non-prescribed antimicrobial use is high (38.8%) [11]. Other similar studies from various countries support this irrational use of medicine, in India (50%–66.7%) [12, 13], Saudi Arabia (48%–77.6%) [14, 15], Uganda (75.7%) [16], Zambia (97%) [17] and in Ethiopia ranges from (14.5% - 43.24%) [18–20].

Several studies evidenced that a number of risk factors for non-prescribed antimicrobial have been documented across the globe. Socio-demographic characteristics including; sex in Northern Uganda [16], Sudan [21], India [22] and Spain [23], age [21, 24–27], marital status [24, 25, 28], occupation [24, 29], income [21, 24], religion [24], residence [25], and educational status [21, 27, 30] have been documented as determinants of non-prescribed anti-microbial drugs use.

Chronic diseases [29], the severity of illness [11, 31–34] and repeated exposure to disease [35] were investigated as risk factors of non-prescribed anti-microbial use. Other important variables such as previous experience with antibiotics [36], waiting time in health facilities and distance of health care facility [37], knowledge-related factors (drug leaflets, awareness, adverse effect, previous exposure and past successful anti-microbial drug use) [11, 15, 16, 20, 31, 33] were identified as risk factors to non-prescribed anti-microbial drugs use. Abuse or misuse of antimicrobials causes considerable public health problems.

Almost all studies conducted in various countries and settings have focused on self-medication. Hence, measuring the magnitude of non-prescribed antimicrobial use (both self and caregiver medications) and its predictors could serve as points of intervention for the concerned bodies. Thus, this study was done to assess the magnitude of non-prescribed anti-microbial use and associated factors among customers in drug retail outlet in Central Zone, Tigray, Ethiopia.

## Methods

### Study design, setting and population

Drug retail outlet based cross-sectional study was conducted from October 1 to November 30/2016 among adults aged 18 years and above in the Central Zone of Tigray, Ethiopia. The Central Zone of Tigray covers area size of 9741 km^2^, and this Zone is administratively divided into nine districts and three major towns (Aksum, Adwa and Abyi Aadi). According to the 2007 population and housing census conducted by Central Statistical Agency of Ethiopia, the total population of Central Zone of Tigray was estimated to be 1,245,824. Of these 613,797 (49.3%) were males, 632,027 (50.7%) were females and 176,453 (14.2%) were urban inhabitants [38]. There are 1 zonal hospital, 1 referral hospital, 2 district hospitals, 57 health centers and around 53 drug retail outlets in the central zone. Adults aged 18 years and above living in Central Zone of Tigray were the source population of the study.

### Sample size determination, sampling techniques and procedures

The sample size was computed using OpenEpi version 2.3 using the assumptions for a single population proportion formula; 43.24% prevalence of self-medication among health sciences students from a study by Gutema et al. in Mekelle University [18], 95% confidence interval, 5% margin of error, design effect of 2 and 10% non-response rate. The final computed sample size was 829**.** The study area was stratified into town and rural districts administrations (‘woredas’ in local administrative structure), and then two towns and three rural districts were selected by simple random sampling technique. Secondly, nine drug retail outlets from town and five drug retail outlets from rural district administrations were also selected using simple random sampling. The study employed proportional to sample size technique and finally, study participants were selected and recruited using consecutive sampling technique.

Customers who received antimicrobial drugs during the data collection period in the selected drug retail outlets were included in this study; whereas those who didn’t live in the study area for at least 6 months were excluded from the study.

### Operational Definitions

#### Antimicrobial

The drugs are provided for the treatment of bacteria, parasites, viruses and fungi, including antibiotics, anti-parasitic, antifungal and anti-viral.

#### Non-prescribed antimicrobial use

Non-prescription-based inappropriate antimicrobial use for treatment of common infections without consulting a medical practitioner and any medical supervision.

#### Care giver medication practice

Non-prescription antimicrobial offers for other sick person to treat health problems of minors or other care seeking with perceived similar health problem.

#### Customers

Clients aged 18 years and above who received antimicrobial from private drug retail outlets.

#### Knowledgeable

When customers’ knowledge was above the computed mean of the ten dichotomized (yes/no) questions, otherwise not knowledgeable.

#### Drug retail outlet

A place used for the conduct of the drug sale, administering or dispensing and licensed by the responsible body as a place wherein the practice of pharmacy may lawfully occur.

#### Systemic infection

Pharmacy personnel diagnoses clinically the respondents’ self-reporting of symptoms of the whole body or disease for which antimicrobial medicines are used.

#### Systemic antibiotics

Are antimicrobials whose effects are manifested to the whole body.

#### Waiting time

The length of time from when the patient entered the outpatient clinic/department to the time the patient actually leaves the OPD (**Fast** = less than 1 h, **Moderate** = 1–2 h, and **Delayed** = more than 2 h).

### Data collection tool and method

A pre-tested and structured questionnaire was employed. This tool was administered in Tigrigna (local language). The questionnaire included socio-demographic characteristics, disease, and medication, knowledge of antimicrobial drugs and its use and health care services. Interviewer- administered was used to collect the data. Fourteen druggists for data collection and three pharmacists for supervision were recruited and these were given two consecutive days training. Per the schedule, principal investigator had supervised and conducted certain meeting during the data collection period.

### Data processing and analysis

Data were entered into EpiInfo version 3.5.4 and exported to SPSS version 21 software package. Coding and cleaning of data were performed for completeness and consistency. Descriptive statistics, frequencies with percentages were computed for categorical and median with Inter-Quartile Range (IQR) for non-normal continuous variables. In the present study, the outcome variable was non-prescribed antimicrobial use, which was dichotomized by assigning 1 for those who received the antimicrobial without prescription and 0 for those who received the antimicrobial with prescription during the data collection time. The binary logistic regression model was used to model the association between outcome and independent variables. Odds Ratio (OR) with 95% confidence interval (CI) was estimated to see the unadjusted (COR) and adjusted (AOR) effect of each factor on non-prescribed antimicrobial use. The candidate independent variables for multivariable analysis were selected at *p*-value of less than 0.20. The level of significance in multivariable analysis level was declared at *P*-value <0.05. The Hosmer and Lemeshow goodness-of-fit, and Omnibus tests of model coefficients were 0.35 and 0.00 respectively. The independent variables were tested for multicollinearity before entering them into the multivariable model, using the Variance Inflation Factor (VIF) test, the Tolerance test, and values of the standard error and acted accordingly.

## Results

### Socio-demographic characteristics

From 829 study sample, a total of 780 respondents participated in this study with a response rate of 94.1%. Out of 780 respondents, 367 (47.10%, 95% CI: 43.80, 50.50) had used non-prescribed antimicrobial during the study period. Nearly two-thirds (67.8%) of respondents who used non-prescribed antimicrobial were males. The median age of non-prescribed antimicrobial users was 30 years (IQR = 16). Out of these respondents who received non-prescribed antimicrobial, 149 (40.6%) were in the age group of 18 to 27 years. Out of these 367 non-prescribed antimicrobial users, 91.8, 79.8, 72.5 and 62.1% were Tigrians in ethnicity, Orthodox-Christians, urban and married, respectively. Pertaining to the educational and occupational status, 121 (33%) and 94 (25.6%) respondents who used non-prescribed antimicrobial were attended secondary school and paid employed, respectively. The median family size of non-prescribed antimicrobial users was 2 (IQR = 1). Nearly three-fifths, 218 (59.4%) of non-prescribed antimicrobial users had a monthly income of ≥32.51 US Dollar (Table 1).

**Table 1:** Socio-demographic characteristics of antimicrobial users in drug retailer outlets in Central Zone, Tigray, Ethiopia, 2016

Characteristics	Antimicrobial Use		
	Prescribed *n* (%)	Non-prescribed *n* (%)	Total *n* (%)
Sex			
Male	224 (54.2)	249 (67.8)	473 (60.6)
Female	189 (45.8)	118 (32.2)	307 (39.4)
Age group (years)			
18–27	131 (31.7)	149 (40.6)	280 (35.9)
28–37	139 (33.7)	101 (27.5)	240 (30.7)
38–47	73 (17.7)	53 (14.4)	126 (16.1)
48–57	37 (9)	29 (7.9)	66 (8.4)
≥ 58	33 (8)	35 (9.5)	68 (8.7)
Ethnic Group			
Tigrians	379 (91.8)	337 (91.8)	716 (91.8)
Others^a^	34 (8.2)	30 (8.2)	64 (8.2)
Religion			
Orthodox-Christian	321 (77.7)	293 (79.8)	614 (78.7)
Muslim	85 (20.6)	65 (17.7)	150 (19.2)
Others^a^	7 (1.7)	9 (2.5)	16(2.1)
Residence			
Urban	329 (79.7)	266 (72.5)	595 (76.2)
Rural	84 (20.3)	101 (27.5)	185 (23.8)
Marital status			
Married	288 (69.7)	228 (62.1)	516 (66.1)
Unmarried	104 (25.2)	119 (32.4)	223 (28.6)
Divorced/widowed	21 (5.1)	20 (5.4)	41 (5.3)
Educational status			
No education	77 (18.6)	75 (20.4)	152 (19.4)
Primary	78 (18.9)	71 (19.3)	149 (19.1)
Secondary	139 (33.7)	121 (33)	260 (33.3)
Tertiary	119 (28.8)	100 (27.2)	219 (28.0)
Occupation			
Unemployed	50 (12.1)	43 (11.7)	93 (11.9)
House wife or farmer	124 (30)	100 (27.2)	224 (28.7)
Student	44 (10.7)	51 (13.9)	95 (12.1)
Self employed	84 (20.3)	79 (21.5)	163 (20.9)
Paid employed	111 (26.9)	94 (25.6)	205 (26.3)
Family size			
≤3	171 (41.4)	159 (43.3)	330 (42.3)
4 to 6	190 (46)	163 (44.4)	353 (45.2)
≥ 7	52 (12.6)	45 (12.3)	97 (12.4)
Monthly income (US Dollar)			
< 6.51	54 (13.1)	76 (20.7)	130 (16.6)
6.51–19.48	51 (12.3)	41 (11.2)	92 (11.7)
19.49–32.5	49 (11.9)	32 (8.7)	81 (10.3)
≥ 32.51	259 (62.7)	218 (59.4)	477 (61.1)

Others^a^: Amhara, Oromo, Guragi

### Types of illnesses reported, non-prescribed antimicrobial use and its mechanism

Out of 367 non-prescribed antimicrobial users, 137 (37.3%) of respondents were given for respiratory system diseases. Three hundred thirty three (90.7%) of the respondents presented to the drug retail outlets with less than or equal to 1-month duration of illness. Two hundred thirty-seven (64.6%) of the non-prescribed antimicrobial were systemic antibiotics followed by 43 (11.7%) for ophthalmic/otic antibiotic. Regarding the mechanism of antimicrobial receiving, among these non-prescribed antimicrobial users, more than half (56.1%) of them got it by telling sign and symptoms of the illness or by providing of a written piece of paper without any signature (Table 2).

**Table 2:** Type of illness reported, anti-microbial drug received and its mechanism among customers in drug retailer outlets in Central Zone, Tigray, Ethiopia, 2016

Illness and antimicrobial	Antimicrobial use		
	Prescribed *n* (%)	Non-prescribed *n* (%)	Total, *n* (%)
Illness^a^			
Respiratory system disease	158 (38.3)	137 (37.3)	295 (37.8)
Gastrointestinal system disease	94 (22.8)	93 (25.3)	187 (24)
Sexual transmitted disease	39 (9.4)	7 (1.9)	46 (6)
Eye disease	31 (7.5)	40 (10.9)	71 (9.2)
Ear disease	12 (2.9)	15 (4.1)	27 (3.4)
Headache	5 (1.2)	7 (1.9)	12 (1.5)
Skin disease, wound	50 (12.1)	40 (10.9)	90 (11.5)
Menstruation and related disease	8 (1.9)	2 (0.5)	10 (1.2)
Malaria	16 (3.9)	26 (7.1)	42 (5.4)
Group of illnesses			
Systemic infection	320 (77.5)	272 (74.1)	592 (76)
Topical infection	50 (12.1)	40 (10.9)	90 (11.5)
Eye/Ear infection	43 (10.4)	55 (15)	98 (12.5)
Duration of illness			
≤ 1 month	381 (92.3)	333 (90.7)	714 (91.5)
> 1 month	32 (7.7)	34 (9.3)	66 (8.5)
Antimicrobial drugs received			
Systemic antibiotic	294 (71.2)	237 (64.6)	531 (68)
Topical antibiotic	19 (4.6)	20 (5.4)	39 (5)
Ophthalmic/Otic antibiotic	38 (9.2)	43 (11.7)	81 (10.4)
Systemic antifungal	5 (1.2)	7 (1.9)	12 (1.5)
Topical antifungal	3 (0.7)	2 (0.5)	5 (0.6)
Anti-protozoan	44 (10.7)	41 (11.2)	85 (11)
Anti-helmethics	10 (2.4)	17 (4.6)	27 (3.5)
Mechanism of antimicrobial receiving			
Provision of prescription	408 (98.8)	5 (1.4)	413 (53)
Naming/Providing drugs/Derivatives/Container/Shape	3 (0.7)	156 (42.5)	159 (20.4)
Telling sign and symptoms or Provision of written piece of paper^b^	2 (0.5)	206 (56.1)	208 (26.6)

Illness^a^ was diagnosed clinically on subjective basis

Written piece of paper^b^: Unsigned paper given by any individual who have some knowledge regarding the medicines

### Antimicrobial and health care service use

Of the 367 non-prescribed antimicrobial users, 326 (88.8%) respondents had visited health facilities for seeking health care in their life; of these, 53.7% received the service from public health facilities, and 175 (53.7%) of these respondents received the service from public health care facilities. Three hundred five (83.1%) of non-prescribed antimicrobial users reported that the respective public health care facilities were found within 5 km radius from their home. Regarding respondents perception of waiting time in health care facilities, 183 (49.9%) and 117 (31.9%) of them reported that they had been served with moderate and delayed waiting time, respectively. Concerning antimicrobial use practice, 280 (76.3%) and 221 (60.2%) of the current non-prescribed antimicrobial users had reported a history of exposure to antimicrobial and non-prescribed antimicrobial use in their life, respectively. One hundred sixty nine (46%) of non-prescribed antimicrobial users had received 1–3 times non-prescribed antimicrobial in the last 6 months. More than half, 152 (54.3%) of non-prescribed antimicrobial users were ever experiencing disease/symptoms in the last 6 months. Out of these 367 respondents who reported non-prescribed antimicrobial use, 230 (62.7%) of them received the drugs for self-medication (Tables 3 and 4).

**Table 3:** Health care services use among customers in drug retailer outlets in Central Zone, Tigray, Ethiopia, 2016

Health Care Services Use	Antimicrobial use		
	Prescribed, *n* (%)	Non-prescribed, *n* (%)	Total *n* (%)
Ever used modern health care services			
No	41 (9.9)	41 (11.2)	82 (10.6)
Yes	372 (90.1)	326 (88.8)	698 (89.4)
Place of receiving modern health care services			
Public health facilities	185 (49.7)	175 (53.7)	358 (51.3)
Private pharmacy/drug store	160 (43.0)	135 (41.3)	295 (42.3)
Others^a^	29 (7.8)	16 (4.9)	45 (6.4)
Distance of health facility from home			
Within 5 km radius	362 (87.7)	305 (83.1)	667 (85.5)
Far from 5 km radius	51 (12.3)	62 (16.9)	113 (14.5)
Self perceived of waiting time in health care facilities			
Fast	119 (28.8)	67 (18.3)	186 (23.8)
Moderate	202 (48.9)	183 (49.9)	385 (49.4)
Delay	92 (22.3)	117 (31.9)	209 (26.8)

Others^a^: Private clinic, NGO

**Table 4:** Antimicrobial use among customers in drug retailer outlets in Central Zone, Tigray, Ethiopia, 2016

Antimicrobial use	Antimicrobial use		
	Prescribed, *n* (%)	Non-prescribed, *n* (%)	Total *n* (%)
Ever received antimicrobial			
No	129 (31.2)	87 (23.7)	216 (26.2)
Yes	284 (68.8)	280 (76.3)	564 (73.8)
Ever received non-prescribed anti-microbial			
No	291 (70.5)	146 (39.8)	437 (56.0)
Yes	122 (29.5)	221 (60.2)	343 (44.0)
Decision on non-prescription antimicrobial drug use (*n* = 344)			
The illness was accidental	33 (27)	46 (20.7)	79 (10.1)
Mildness of illness	26 (21.3)	37 (16.7)	63 (8.1)
The drug was a broad anti-microbial	15 (12.3)	21 (9.5)	36 (4.6)
Treated with the previous drug	24 (19.7)	48 (21.6)	72 (9.2)
Less money and time consumed	22 (18)	68 (30.6)	90 (11.5)
Others	2 (1.6)	2 (0.9)	4 (0.5)
In the past 6 months, how often non-prescription anti-microbial drug has been purchased			
None	145 (35.1)	82 (22.3)	227 (29.1)
1–3	121 (29.3)	169 (46)	290 (37.1)
≥ 4	147 (35.6)	116 (31.6)	263 (33.8)
In the past 6 months, ever experienced similar disease/symptoms (*n* = 564)			
No	163 (57.4)	128 (45.7)	291 (51.6)
Yes	121 (42.6)	152 (54.3)	273 (48.4)
For whom does this antimicrobial drug provide			
Myself	289 (70)	230 (62.7)	519 (66.5)
Father/mother/brother/sister	39 (9.4)	46 (12.5)	85 (10.9)
Daughter/son	78 (18.9)	83 (22.6)	161 (20.6)
Others (wife, husband etc.)	7 (1.7)	8 (2.2)	15 (2)
Have you received this non-prescribed anti-microbial for:			
Self-medication	289 (70)	230 (62.7)	519 (66.5)
Caregiver medication	124 (30)	137 (37.3)	261 (33.5)
Magnitude of non-prescribed antimicrobial use	-	367 (47.1)	-

### Knowledge on antimicrobial use

The magnitude of non-prescribed antimicrobial use was 47.1% (95% CI: 43.80, 50.50). Of the total respondents included in this study, 741 (95.0%) respondents knew that antimicrobial cannot be taken with alcohol. Similarly, 719 (92.1%) of respondents knew that placing of antimicrobial in a safe and unreachable area to children have benefits. More than three-fourth (79.9%) of respondents reported that drug resistance can occur when antimicrobial users do not complete the full course of treatment in the prescribed dose. Out of the total respondents, 697 (89.3%) of them knew that antimicrobial prescribed for adults should not be given to children, and 703 (90.1%) of them knew that all antimicrobial do not be given to pregnant and lactating mothers. Four hundred sixty four (59.5%) of respondents knew that the expiry date of the antimicrobial should be checked before using it. Based on the composite mean score of knowledge, 405 (51.9%) of the respondents were knowledgeable regarding to antimicrobial use (Table 5).

**Table 5:** Knowledge of antimicrobial among customers in drug retailer outlets in Central Zone, Tigray, Ethiopia, 2016

Knowledge of Antimicrobial Drug use	Use of Antimicrobial drug(s)		
	Prescribed *n* (%)	Non-prescribed *n* (%)	Total *n* (%)
Anti-microbial with some other drugs			
No	214 (51.8)	159 (43.3)	373 (47.9)
Yes	199 (48.2)	208 (56.7)	407 (52.1)
Anti-microbial with alcohol			
No	389 (94.2)	352 (95.9)	741 (95.0)
Yes	24 (5.8)	15 (4.1)	39 (5.0)
Ant-microbial with some foods^a^			
No	187 (45.3)	167 (45.5)	354 (54.6)
Yes	226 (54.7)	200 (54.5)	426 (45.4)
Benefit from placing of anti-microbial at unreachable area to children			
No	42 (10.2)	19 (5.2)	61 (7.9)
Yes	371 (89.8)	348 (94.8)	719 (92.1)
Anti-microbial resistance when incomplete the prescribed dose			
No	84 (20.3)	73 (19.9)	157 (20.1)
Yes	329 (79.7)	294 (80.1)	623 (79.9)
Anti-microbial prescribed for adult sharing to children			
No	364 (88.1)	333 (90.7)	697 (89.3)
Yes	49 (11.9)	34 (9.3)	83 (10.7)
All types of anti-microbial give to pregnant and lactating mothers			
No	367 (88.9)	336 (91.6)	703 (90.1)
Yes	46 (11.1)	31 (8.4)	77 (9.9)
All types of anti-microbial give to patients with chronic diseases			
No	341 (82.6)	304 (82.8)	645 (82.7)
Yes	72 (17.4)	63 (17.2)	135 (17.3)
Prescribed anti-microbial rightly use according the dispenser information			
No	85 (20.6)	80 (21.8)	165 (21.2)
Yes	328 (79.4)	287 (78.2)	615 (78.8)
Checking expiry date of the anti-microbial			
No	155 (37.5)	161 (43.9)	316 (40.5)
Yes	258 (62.5)	206 (56.1)	464 (59.5)
Overall knowledge status (composite mean score = 14.6)			
Not knowledgeable	203 (49.2)	172 (46.9)	375 (48.1)
Knowledgeable	210 (50.8)	195 (53.1)	405 (51.9)

Foods^a^: milk, egg

### Factors associated with non-prescribed antimicrobial use

In the bivariate analysis, sex, age group, residence, marital status, monthly income, group of illnesses, place of seeking modern healthcare services, distance of health facility from home, self perceived of waiting time in health care facilities, ever received antimicrobial drug and frequency of non- prescribed antimicrobial drugs purchased in the past 6 months were associated with non-prescribed antimicrobial use.

The multivariable analysis being male, receiving health care services from private/NGO, moderate waiting time, delayed waiting time, ever received antimicrobial in life, 1–3 times and ≥4 times frequency of purchasing of antimicrobial in the last 6 months were independently associated factors with non-prescribed antimicrobial use.

Being male [AOR = 1.72, 95% CI = 1.21, 2.44] was about 1.72 times more likely to have non-prescribed antimicrobial use compared to their counterparts. Receiving health care services from private/NGO [AOR = 0.47, 95% CI; 0.23, 0.98] was about 53% less likely to use non-prescribed anti-microbial than those who received the services from public health facilities. Perceived waiting time in health care facilities was also associated with non-prescribed antimicrobial use; respondents who reported that moderate waiting time [AOR = 1.92, 95% CI; 1.20, 3.09] and delayed waiting time [AOR = 1.56, 95% CI; 1.03, 2.38] were almost two folds and 1.56 times, respectively more likely to use non-prescribed antimicrobial compared to those who reported fast waiting time in health care facilities.

Respondents who ever have received antimicrobial were nearly four times to have non-prescribed anti-microbial use compared to those who never received antimicrobial in life, [AOR = 3.51, 95% CI; 2.45, 5.02].

Regarding the frequency of purchasing non-prescribed antimicrobial in the past 6 months, those respondents who purchased 1–3 times [AOR = 2.04, 95% CI; 1.36, 3.06] and who purchased ≥4 times (AOR = 2.66, 95% CI; 1.24, 5.68) in the last 6 months were 2.04 and 2.66 times, respectively more likely to currently practice non-prescribed antimicrobial use than those who never have used non-prescribed antimicrobial at this period (Table 6).

**Table 6:** Bivariable and multivariable logistic regression analysis of factors associated with non-prescribed antimicrobial use among customers in drug retailer outlets in Central Zone, Tigray, Ethiopia, 2016

Characteristics	Use of Antimicrobial drug(s)		COR (95% CI)	AOR (95% CI)
	Prescribed, *n* (%)	Non-prescribed, *n* (%)		
Sex				
Male	224 (54.2)	249 (67.8)	1.78 (1.33, 2.39)^b^	1.72 (1.21, 2.44)^b^
Female	189 (45.8)	118 (32.2)	1	
Age group (years)				
18–27	131 (31.7)	149 (40.6)	1	
28–37	139 (33.7)	101 (27.5)	0.64 (0.45, 0.90)^b^	
38–47	73 (17.7)	53 (14.4)	0.64 (0.42, 0.98)^b^	
48–57	37 (9)	29 (7.9)	0.69 (0.40, 1.18)^a^	
≥ 58	33 (8)	35 (9.5)	0.93 (0.55, 1.59)	
Residence				
Urban	329 (79.7)	266 (72.5)	1	
Rural	84 (20.3)	101 (27.5)	1.49 (1.07, 2.07)^b^	
Marital status				
Married	288 (69.7)	228 (62.1)	1	
Unmarried	104 (25.2)	119 (32.4)	1.45 (1.05, 1.98)^b^	
Divorced/Widowed	21 (5.1)	20 (5.4)	1.20 (0.64, 2.27)	
Monthly income (US Dollar)				
< 6.51	54 (13.1)	76 (20.7)	1.67 (1.13, 2.48)^b^	
6.52–19.48	51 (12.3)	41 (11.2)	0.96 (0.61, 1.50)	
19.49–32.50	49 (11.9)	32 (8.7)	0.78 (0.48, 1.26)	
≥ 32.51	259 (62.7)	218 (59.4)	1	
Group of Illness				
Systemic Infection	320 (77.5)	272 (74.1)	1	
Topical Infection	50 (12.1)	40 (10.9)	0.94 (0.60, 1.47)	
Eye/Ear Infection	43 (10.4)	55 (15)	1.51 (0.98, 2.31)^a^	
Receiving modern health care services				
Public health facilities	184 (49.3)	176 (53.8)	1	1
Private pharmacies/drug stores	160 (42.9)	135 (41.3)	0.88 (0.65, 1.20)	-
Others (Private clinic, NGO)	29 (7.8)	16 (4.9)	0.58 (0.30, 1.09)^a^	0.47 (0.23, 0.98)
Distance of health facility from home				
Within 5 km radius	362 (87.7)	305 (83.1)	1	
Far from 5 km radius	51 (12.3)	62 (16.9)	1.44 (0.97, 2.15)^a^	
Self perceived of waiting time in health care facilities				
Fast	119 (28.8)	67 (18.3)	1	1
Moderate	202 (48.9)	183 (49.9)	2.26 (1.51, 3.39)^b^	1.92 (1.20, 3.09)^b^
Delay	92 (22.3)	117 (31.9)	1.61 (1.12, 2.31)^b^	1.56 (1.03, 2.38)
Ever received antimicrobials				
No	129 (31.2)	87 (23.7)	1	1
Yes	284 (68.8)	280 (76.3)	1.46 (1.06, 2.01)^b^	3.51 (2.45, 5.02)^b^
Ever received non-prescription antimicrobials				
No	291 (70.5)	146 (39.8)	1	
Yes	122 (29.5)	221 (60.2)	3.61 (2.68, 4.86)^b^	
In the past 6 months, how often non- prescription anti-microbial purchased				
None	145 (35.1)	82 (22.3)	1	1
1–3	121 (29.3)	169 (46)	2.47 (1.73, 3.53)^b^	2.04 (1.36, 3.06)^b^
≥ 4	147 (35.6)	116 (31.6)	1.39 (0.97, 2.01)^a^	2.66 (1.24, 5.68)

Bivariable ^b^at α < 0.05, ^a^at α < 0.20 and multi variable ^b^significant at < 0.01, US Dollar = United State Dollar

## Discussion

This study has assessed the magnitude and factors associated with non-prescribed antimicrobial use. As a result, the magnitude of non-prescribed antimicrobial use was 47.1%, and the independently associated factors with non-prescribed antimicrobial use were being male, receiving health care services from private/NGO, moderate waiting time, delayed waiting time, ever received antimicrobial in life, 1–3 times and ≥4 times frequency of purchasing of antimicrobial in the last 6 months.

The magnitude of non-prescribed antimicrobial use reported by this study is in line with studies conducted in various countries across the world; in Ethiopia (43.24%), [18], in Yemen, Saudi Arabia, and Uzbekistan (48%) [15] and in Northern India (50%) [13]. However, it is higher than the findings reported from southwestern Ethiopia (39%) [20], South Ethiopia (14.5%) [19], in a systematic and meta-analysis study in low and middle income countries, the overall estimate of antimicrobial use is 38.8% [11] and Saudi Arabia (35.4%) [26]. The finding of this study is lower than findings documented in several studies; in northern Uganda (75.7%) [16], in Zambia (97%) [17], in Bangalore, India (66.7%) [12] and in Riyadh, Saudi Arabia (77.6%) [14]. The possible explanation could be due to these studies varying in the nature of definitions used; the present study used non-prescribed antimicrobial (both self and caregiver medication practices) whereas these studies included as comparison with the current finding applied only self-medication as outcome variable. Regarding to recall period considered for definition, in this study, respondents were interviewed whether received non-prescribed antimicrobial or not at time of data collection period. However, studies conducted in Riyadh, Saudi Arabia, respondents were asked about practicing of self-medication in the last 2 weeks and in Sudan, 1–2 months. Additionally, the observed discrepancy could be due to geographical variation of the region selected, methodology adopted and the characteristics of study populations.

In the present study male sex was 1.7 times more likely to practice non-prescribed antimicrobial use compared to their female counterparts. Similar findings have been evidenced in Northern Uganda [16], in Indonesia [39], in Urban Puducherry, India [22], in Yogyakarta City Indonesia [28], in Riyadh, Saudi Arabia [26], in Sudan [40]. This finding is contrary to other studies done in Sudan [21], in Spain [23] and South India [32]. This difference may be due to in the current study context, males have a better health seeking behavior compared to females.

In this study those respondents who previously received health care services from private clinic/NGOs were less likely to practice non-prescribed antimicrobial compared to those who received health care from public health facilities. This finding is in line with study conducted in Riyadh, Saudi Arabia [26]; reporting inconvenient access or dissatisfied with public health care were more likely to practice self-medication. The above finding might be also related with longer waiting time in health facilities effect on perception of respondents for consuting physicians for every health complaints. In this study, perceived moderate and delayed waiting time in health care facilities was documented as a risk factor for practicing of non-prescribed antimicrobial compared to perceived fast waiting time. Even though less literature available on the relationship between treatment-seeking behavior and self-medication or self-care in the populations of developing countries [41]. Studies in Tanzania and Sudan also revealed that reasons for self-medication were shortages of drugs, long waiting time, long distance at/to health facilities, inability to pay for health care charges and the freedom to choose the preferred drugs [33, 42, 43].

Respondents who ever have received antimicrobial before the study period were more likely to use non-prescribed antimicrobial currently than their counterparts. This is in line with the finding of similar study in University of Gondar, Ethiopia [31], in post-conflict Northern Uganda [16] and in a multi-center study in five cities of Pakistan revealed that prior or previous experience was a predictor of self-medication [41].

Moreover, pertaining to frequency of purchasing non-prescribed antimicrobials; those who purchased non-prescribed antimicrobials 1–3 times and greater or equal to four times in the past 6 months were statistically associated with non-prescribed anti-microbial use. Similar finding was documented in rural China that revealed purchasing antibiotics without physicians prescription has effect on behavior of self-medicating children with antibiotics [44].

This study has its own limitations as it did not consider attitude variable, and possible availability of social desirability bias might have a negative effect on the findings of the study.

## Conclusion

The magnitude of non-prescribed antimicrobial use was high. Male sex, receiving health care services from private/NGO, waiting time in health care facilities, ever received antimicrobial, and frequency of purchasing non-prescribed anti-microbial was independently associated with non-prescribed antimicrobial use. Community education through the involvement of the private health sector and health care providers should be given an emphasis. Regulation and policy enforcement are necessary to promote the rational use of antimicrobial.

## Abbreviations

AOR: Adjusted Odds Ratio
CI: Confidence Interval
COR: Crude Odds Ratio
IQR: Inter Quartile Range
LMICs: Low and Middle Income Countries
NGOs: Non-Governmental Organizations
WHO: World Health Organization
